# Knowledge on Newborn Life Support among the Healthcare Providers in a Tertiary Care Maternity Hospital in the Southern Province, Sri Lanka

**DOI:** 10.1155/2021/6991584

**Published:** 2021-11-28

**Authors:** N. D. Liyanarachchi, B. H. H. Pradeepa

**Affiliations:** ^1^Faculty of Medicine, University of Rununa, Matara, Sri Lanka; ^2^Faculty of Allied Health Science, University of Ruhuna, Matara, Sri Lanka

## Abstract

**Introduction:**

The newborn life support (NLS) is a set of educational guidelines established by the academies of Paediatrics that outline the proper procedures for resuscitation of a newborn. The objective of this study was to determine the knowledge on NLS among the healthcare providers (HCPs) in a tertiary care maternity hospital in the Southern Province, Sri Lanka.

**Methods:**

A hospital-based cross-sectional study was carried out among doctors, nurses, and midwives, using a self-administered questionnaire. Comparison of knowledge among different categories was made using the chi-square test. Total sample of 191 consisted of 118 (61.8%) nurses, 33 (17.3%) midwives, and 36 (18.8%) doctors. The majority of HCPs (76.7%) had good knowledge of NLS; however, following guidelines on NLS among HCPs was poor (33%). According to the category, 91% of doctors and 78% of nurses had good knowledge, whereas only 48% of midwives had good knowledge. The difference of knowledge on NLS among different categories of HCPs was statistically significant (*p* < 0.001). Only 33% of HCPs had good knowledge of following NLS guidelines. Of them, 52% were doctors, 31% were nurses, and only 18% were midwives. The difference in adherence to NLS guidelines among different categories of HCPs was highly significant statistically (*p*=0.003).

**Conclusion:**

The majority of the healthcare providers had good knowledge of NLS. There was a significant difference in the level of knowledge on NLS among different categories of HCPs. Gaps in the knowledge in following guidelines of NLS were noted in the majority. Newborn resuscitation has to be included in nursing and midwifery curricula, and training on NLS is essential in the orientation programs for newly recruited HCPs in maternity hospitals.

## 1. Introduction

Effective newborn resuscitation is essential for reducing the adverse outcomes of birth asphyxia [1]. Most newborn deaths are associated with birth asphyxia (40%), low birth weight and prematurity (25%), and infections (20%) [2]. The global average contribution of neonatal mortality to under-five mortality is 47%, of which 11% accounts for perinatal asphyxia [3]. Based on a recent systematic review, about one-third of all neonatal deaths occur during the first 24 h of birth, and close to three quarters die in the first week of life [4]. These findings suggest that focusing on the critical periods before and immediately following birth is essential to saving more newborn lives. In Sri Lanka, the neonatal mortality rate has declined to 7/1000 live births by 2019 [5]. Yet, it accounts for over 70% of under-five mortalities of our children [6].

Newborn resuscitation is an interventional procedure used to assist the airway, breathing, and circulation at birth [7]. Many medical professionals, especially those dealing directly with newborns, must complete the NLS course [8]. It has been shown that providing basic training on resuscitation of newborns can decrease neonatal deaths [1]. In the US, the Neonatal Resuscitation Program (NRP) is the primary educational mechanism used to teach healthcare providers (HCPs) to perform neonatal resuscitation [9]. The goal of the NRP is to help neonatal care providers (NCPs) acquire the cognitive, technical, and behavioral skills needed for successful and efficient resuscitation of babies at the time of birth [9]. Sri Lanka College of Paediatricians (SLCP) developed newborn life support (NLS) and initiated a NLS course for the first time in Sri Lanka in September 2006 at the Lady Ridgeway Hospital for Children, Colombo, under the guidance of the Resuscitation Council of UK [7, 8]. Family Health Bureau (FHB) of Ministry of Health, Sri Lanka, SLCP, and Perinatal Society of Sri Lanka are conducting courses on NLS island-wide to train doctors, nurses, and midwives working in the maternity hospitals. Most of the HCPs working in the maternity units of tertiary care hospitals of Sri Lanka have undergone training on NLS.

Studies regarding the level of knowledge among healthcare providers on NLS are sparse in Sri Lanka. A study done at Kandy, Sri Lanka, by Ralapanawa et al. has demonstrated a good mean knowledge score of 67.6% on basic life support among doctors and medical students [10].

To the best of our knowledge, only limited studies have been published globally regarding the level of knowledge among healthcare providers on NLS [11–13].

This study aimed to determine NLS knowledge among the healthcare providers (HCPs) in Teaching Hospital Mahamodara (THM), Galle, Sri Lanka. Midwives and nurses are the first to come in contact with a newborn in the delivery suite. Therefore, assessing the knowledge among them and the doctors is important in improving neonatal care. The findings of this study could be used to identify the gaps in the knowledge of HCPs on NLS. It could provide important clues and insight to design evidence-based tailor-made interventions like training programs and workshops to improve the HCP's knowledge and practice of NLS. Aiming to reduce the neonatal mortality rate (NMR) is an objective of the millennium goals of the WHO. Birth asphyxia contributes to NMR [3]. Such intervention would help to reduce neonatal mortality and morbidity in the country.

## 2. Methods

This hospital-based cross-sectional study was carried out in THM, Galle, Sri Lanka. THM has an average birth rate of over 10,000 per year, and the number of HCPs involved in neonatal care was 191. HCPs consisting of medical officers, nursing officers, and midwives working for more than three months at THM were enrolled in the study.

Data collection was done using a self-administered questionnaire. It consisted questions to collect social demographic data and knowledge on NLS and its guidelines. The questions to assess the knowledge were prepared based on the NLS manual published by SLCP [8] and a questionnaire that has already been used at Teaching Hospital Peradeniya, Kandy, Sri Lanka, in a similar study [10]. Participants responded to the questionnaire individually during their free time while on duty, and one of the investigators was at the site when the participants answered the questionnaire, and discussions were not allowed among participants. The questionnaire comprised ten questions to check the knowledge and another six questions to determine whether HCPs follow the NLS guidelines laid down by SLCP.

Each question was followed by three responses to select, and a correct response was given +1 mark, and the wrong response was given a zero mark. The level of knowledge was categorized based on the cumulative score. Those who scored nine and above for questions on knowledge were considered to have very good knowledge, whereas scores from 6 to 8 were considered good, scores from 4 to 5 were considered as average, and scores from 0 to 3 were considered as having poor knowledge.

All data were coded and entered into a database created with Microsoft Excel. Data analysis was done using Statistical Package for Social Sciences (SSPS) version 20.0. The descriptive statistics such as mean and percentages (%) were estimated, and the chi-square test was used to compare the knowledge and adherence to NLS guidelines by different categories of HCPs. The level of significance was considered 0.05. Ethical clearance was obtained from the Ethics Review Committee of the Faculty of Medicine, University of Ruhuna (Ref. No. 14.02.2018:033), and administrative clearance was obtained from the Director, THM. Informed written consent was obtained from all participants.

## 3. Results

All 191 employees attended the study (response rate was 100%). The majority of the study population (62%) were nursing officers, and the age ranged from 26 to 59 years (mean ± SD = 37.80 ± 7.98 years). The basic socio-demographic characteristics of the participants are presented in Table 1.

### 3.1. Training on NLS

The HCPs in the study sample were exposed to different training levels on newborn resuscitation, which are listed in Table 2. The majority (90%) of HCPs had been exposed to some form of training on newborn resuscitation. Only 34% of HCPs had followed the formal NLS course conducted by the SLCP. Out of 191 HCPs, 20 (10.5%) have not had any training on newborn resuscitation.

### 3.2. The Knowledge of NLS

The knowledge on NLS was good or very good in 76.5% of HCPs (very good: 14.14%, and good: 62.30%), while 25% of HCPs had average or below-average knowledge. The knowledge scores of the HCPs are demonstrated in Table 3. The majority of doctors and nurses had good knowledge, whereas midwives' knowledge of NLS was not as good as doctors and nurses. The knowledge scores of NLS among the different categories of HCPs are demonstrated in Table 4. The difference of knowledge on NLS among different categories of HCPs was statistically significant (*p* < 0.001).

### 3.3. Adherence to NLS Guidelines

The majority of HCPs (88.5%) thought that they were following NLS guidelines accurately. However, according to NLS guidelines, 67% of them did not score good marks on assessing the knowledge on resuscitation. The relationship between adherence to NLS guidelines and the category of HCPs is shown in Table 5. The difference in the level of knowledge of adherence to guidelines on NLS between categories of HCPs was highly significant statistically (*p*=0.003).

The majority (72.8%) of HCPs perceived that they did not have adequate training on NLS. Only 14.1% of HCPs said that they had got adequate training on NLS during their undergraduate training. A large portion of the study population (94.2%) wanted to acquire further knowledge and skills on NLS to manage cases confidently.

## 4. Discussion

NLS is an essential component of neonatal care services and is an inexpensive intervention by which many newborn lives can be saved. This study assessed NLS knowledge among HCPs in a tertiary care hospital where proper neonatal and obstetrics care are available.

### 4.1. Knowledge on NLS

In the present study, the majority of healthcare providers had good knowledge of NLS. It may be due to the formal and informal training on NLS received by the HCPs from the workplace. The HCPs in our study population are working in a busy tertiary care centre where average daily deliveries are around 25 to 30, and they get adequate exposure on NLS. However, midwives' knowledge was not satisfactory, and only 48% of the midwives had good knowledge on NLS when compared to other categories of HCPs. Several factors would contribute to this finding such as less exposure to NLS during the midwifery training, difficulty in understanding when the NLS course is conducted in common to all HCPs, or less opportunities offered to them. Ralapanawa et al.'s study demonstrated a good mean knowledge score of 67.6% on Advanced Paediatric Life Support among doctors and medical students in Sri Lanka [10]. A significantly higher proportion of final year medical students had good knowledge than medical officers in that study. However, there are no studies found locally regarding assessment of knowledge on NLS among other healthcare providers. A similar result was found in a study done in Nigeria where nurses' knowledge and practice were assessed, and it was found that 78.8% of them had adequate knowledge of newborn resuscitation [11]. In contrast to the above findings, a study done in Kenya indicates that only 35.4% of the participants scored above the minimum knowledge competency level [12]. In two studies done in Ethiopia (knowledge score of 42.8%) [13] and Ghana (knowledge score of 38%) [14], it was found that the overall knowledge on neonatal resuscitation in health professionals was poor. This was thought to be due to lack of exposure to an adequate number of real resuscitation cases, simulation-based training, updating training, and certification process.

Medical officers were found to have better knowledge level regarding NLS than nursing officers and midwives in our study. The difference in the level of knowledge between categories of HCPs was statistically significant (*p* < 0.001). Undergraduate exposure to newborn resuscitation and training before the internship would be the reason for our study's better knowledge of NLS among doctors. The nursing officers and midwives do not receive such training before they enter the services at present. Once HCPs enter the service, all of them receive similar training on NLS irrespective of the category. In contrast to our results, there was no significant difference in the knowledge score of the participants in terms of the type of profession (*p*=0.847) and qualification (*p*=0.055) in a study done in Ethiopia on newborn resuscitation [13]. A cross-sectional study in Afghanistan revealed no significant differences in knowledge, clinical skills, or confidence in performing newborn resuscitation between doctors and midwives [15]. In Afghanistan, newborn resuscitation is considered an essential midwifery competency and has been part of the national midwifery curriculum since 2004 [15].

### 4.2. Training in NLS

Maintenance of resuscitation skills requires knowledge, ongoing practice, and periodic refresher training. Our study found that most HCPs had exposure to some training on NLS during their career, and one-third of HCPs had followed the NLS course. All the categories of HCPs get the similar NLS training after their graduation. One-third of the HCPs in our study group have been exposed to NLS course due to implementing a national program by the SLCP and the FHB of Sri Lanka to train the HCPs in the maternity hospitals on NLS. To the best of our knowledge, no study has been done locally to compare the findings. However, there is not much evidence in the literature that HCPs receive in-service training in NLS in other developing countries. In Tanzania, only 33% of staff reported receiving refresher training on newborn resuscitation [14]. A study conducted in the Philippines found that <50% of staff were trained in neonatal and paediatric resuscitation [16].

Only a very small proportion of HCPs said they had got adequate training on NLS before they started their career. This indicates that little emphasis is given to NLS training in the respective undergraduate curricula in Sri Lanka. The level of knowledge that HCPs received during their undergraduate education and their performance level would have contributed to the difference in perceiving the adequacy of training among the HCPs. A large portion of the study sample wanted training on NLS to improve their knowledge and skills.

### 4.3. Knowledge on Adherence to NLS Guidelines

The majority of HCPs had perceived that they were following NLS guidelines accurately. However, according to the marks they scored in the questionnaire, only one-third of HCPs had good knowledge of following exact steps according to NLS guidelines. The majority were doctors. Only a very low proportion (<20%) of midwives has shown good knowledge on adherence to NLS guidelines. The difference in ability on adherence to NLS guidelines among the three categories of HCPs was highly significant. Although all three types of HCPs are trained on NLS guidelines similarly, there is a statistically significant difference in the knowledge of NLS guidelines among HCPs. This indicates that the method of training NLS guidelines would not suit every HCP category. The very poor knowledge on adherence to NLS in midwives may be due to a language barrier in gathering knowledge and skill of NLS during the training for all HCPs. This is very important because, as the first contact of a delivery suite, the midwives should be trained well to initiate NLS until help is sought from other HCPs. A further evaluation is necessary to conclude in this regard. According to their year of graduation, there is no statistical significance of the knowledge on adherence to NLS guidelines in HCPs. The majority of HCPs wanted to improve their understanding regarding NLS guidelines which shows their readiness to get new knowledge. The present study highlights the need for structured training of NLS for HCPs. In order to optimize newborn life support, adherence to the guidelines is essential. Thus, mortality and morbidity due to birth asphyxia can be brought down. However, the study has some limitations; it was a questionnaire-based study, and the skill of the individuals added to their intellect could not be measured. Hence, it is recommended to conduct further studies on a larger scale to get a better picture.

## 5. Conclusions and Recommendations

The majority of the healthcare providers in our study group had good knowledge of NLS. However, there was a significant difference in the level of knowledge among different categories of HCPs. There were gaps in the knowledge on NLS guidelines in the majority. Mandatory training on NLS needs to be included in the orientation programs of intern medical officers, newly recruited nursing officers, and midwives in maternity hospitals. We recommend incorporating newborn resuscitation in nursing and midwifery curricula as it is vital to train midwives as they are the first to come in contact with a newborn at delivery. Regular refresher training sessions are needed to improve the knowledge and skills of HCPs; thus, the newborn resuscitation process in the maternity units could be improved, leading to a reduction of neonatal morbidity and mortality. Large multicentre studies are needed to confirm the results of this type of preliminary study.

## Figures and Tables

**Table 1:** Socio-demographic characteristics of the HCPs (*n* = 191).

Characteristics	Frequency	Percentage
*Position*		
House officers	10	5.2
Medical officers	19	9.9
Registrars	4	2.1
Senior registrars	3	1.6
Nursing sisters	4	2.1
Nursing officers	118	61.8
Midwives	33	17.3

*Gender*		
Female	172	90.1
Male	19	9.9

*Age*		
34 years or less	68	35.6
35–50 years	90	47.1
51–65 years	14	7.3
Missing	19	9.9

**Table 2:** Method of training on newborn life support received by the HCPs (*n* = 191).

Method of training	Frequency	Percentage
No training	20	10.47
By observing resuscitation done by a trained HCP	22	11.52
Following lectures	58	30.37
Following NLS course	65	34.03
Observing and following lectures	11	5.75
Following lectures and following NLS course	8	4.18
All of the above methods	7	3.66
Total	191	100.00

**Table 3:** Distribution of knowledge score of HCPs on NLS (*n* = 191).

Category of knowledge level	Number	Percentage
Very good (9-10)	27	14.14
Good (6–8)	119	62.30
Average (4-5)	41	21.47
Poor (0–3)	4	2.09
Total	191	100

**Table 4:** Comparison of knowledge on NLS among different categories of HCPs (*n* = 191).

Position	Level of knowledge on NLS				Total	Significance
	9-10 (very good)	6–8 (good)	4-5 (average)	0–3 (poor)		
	No (%)	No (%)	No (%)	No (%)	No (%)	
Medical officer	9 (4.7)	24 (12.5)	3 (1.57)	0 (0)	36 (18.8)	*χ*2 = 18.43
Nursing officer	17 (8.9)	80 (41.8)	22 (11.5)	3 (1.57)	122 (63.8)	df = 3
Midwives	1 (0.5)	15 (7.8)	16 (8.37)	1 (0.52)	33 (17.2)	*p* < 0.001^*∗*^
Total	27 (14.1)	119 (62.3)	41(21.4)	4 (2.09)	191 (100)	

^*∗*^ *p*value is significant at 0.05.

**Table 5:** Adherence to NLS guidelines by category of HCPs (*n* = 191).

Designation	Knowledge on adherence to NLS guidelines			Total	Significance
	5-6 (good)	3-4 (average)	0–2 (poor)		
	No (%)	No (%)	No (%)	No (%)	
Medical officers	19 (9.9)	11(5.7)	6 (3.1)	36 (18.8)	*χ*2 = 8.943
Nursing officers	38(19.8)	65(34.0)	19(9.9)	122 (63.3)	df = 2
Midwives	6 (3.1)	16(8.3)	11 (5.7)	33(17.2)	*p* < 0.003^*∗*^
Total	63(32.9)	92(48.1)	36(18.8)	191(100)	

^*∗*^ *p*value is significant at 0.05.

## Data Availability

The raw data used to support the findings of this study are available from the corresponding author upon request.

## Supplementary Materials

Supplementary Materials Questionnaire in English: this is the self-administered questionnaire used to collect data.
