# Supplementary material for: Knowledge on Newborn Life Support among the Healthcare Providers in a Tertiary Care Maternity Hospital in the Southern Province, Sri Lanka
Source: Nurs Res Pract. 2021 Nov 28;2021:6991584. doi: 10.1155/2021/6991584 (PMC8645392; doi:10.1155/2021/6991584)
Supplement: Supplementary Materials — Questionnaire in English: this is the self-administered questionnaire used to collect data. [file 6991584.f1.docx]

**Knowledge on newborn life support among the health care providers (HCP) in Teaching Hospital Mahamodara.**

1. **Socio- Demographic Data**

1. Age : …………………

2. Sex :

|  |
| --- |
|  |

Male

Female

3. Position :

|  |
| --- |
|  |
|  |
|  |
|  |
|  |
|  |

3.1. Intern H/O

3.2. SHO

3.3. Registrar

3.4. Senior Registrar

3.5. Sister

3.6. Nursing officer

3.4. Midwife

4. In which year did you graduate or complete your diploma …………………

5. In which specialty are you currently working?

|  |
| --- |
|  |
|  |
|  |
|  |

5.1. Obstetric wards

5.2. Labor rooms

5.3. Theatres

5.4. Postnatal wards

5.5. NICU/ PBU

6. Have you ever worked in an NICU/ PBU at least 3 months?

|  |
| --- |
|  |

Yes

No

7. Have you ever had formal teaching on newborn life support? ( Mark with 🗸,

multiple responses are allowed)

|  |
| --- |
|  |
|  |
|  |

- 1. None
  2. Observed only
  3. Lectures
  4. Followed NLS course

1. **Knowledge regarding newborn life support** ( Mark the correct response with a “🗸”)
2. Have you heard about Apgar score?

|  |
| --- |
|  |

Yes

No

1. Mark the parameters used in Apgar?

|  |
| --- |
|  |
|  |
|  |
|  |
|  |

2.1. Color

2.2. Tone

2.3. Blood pressure

2.4. Pulse

2.5. Respiration

2.6. Grimace

1. What is the recommended position of the head for management of Airway in a newborn baby?

|  |
| --- |
|  |
|  |
|  |

3.1. Supine position (Head tilt, chin lift)

3.2. Neutral position

3.3. Prone position

3.4. Do not know.

1. If the baby doesn’t start breathing after keeping the head in the correct position. What would you do?

|  |
| --- |
|  |
|  |

4.1. Give five inflation breaths

4.2. Endo tracheal tube insertion

4.3. Suck out secretion

1. What is the recommended way to determine the location point for chest

compressions?

|  |
| --- |
|  |
|  |

5.1. Lower sternal edge

5.2. Apex of the heart

5.3. Don’t know

1. What is the recommended depth of chest compression?

|  |
| --- |
|  |
|  |

6.1. Depth of 1/3of the chest

6.2. Depth of 1/2 of the chest

6.3. Don’t know

1. What is the recommended ratio of chest compressions to ventilations in NLS?

|  |
| --- |
|  |
|  |

7.1. 15: 2

7.2. 3: 1

7.3. Don’t know

1. What is the rate of chest compression in newborn during Resuscitation?

|  |
| --- |
|  |
|  |

8.1. 100/ min

8.2. 120/ min

8.3. 80/ min

9) The dose of Adrenaline, while managing a baby with anincidence of bradycardia?

|  |
| --- |
|  |
|  |

9.1. 1mg/ kg

9.2. 0.1ml/ kg

9.3. Don’t know

1. What type of tubes use during Endotracheal intubation in neonates?

|  |
| --- |
|  |
|  |

10.1. Cuffed tubes

10.2. Non Cuffed tubes

10.3. Don’t know

**3.Guidelines on newborn life support**

1) How would assess the baby’s condition at birth?

|  |
| --- |
|  |
|  |

1.1. By Apgar score

1.2. By check CTBH

1.3. By check the colour

2) When a baby did not cry at birth, what would you do first?

|  |
| --- |
|  |
|  |

2.1. Keep the baby under a radiant warmer

2.2. Dry the baby and remove wet linen

2.3. Start giving inflation breaths

1. If a baby doesn’t respond to airway opening, what would you do next?

|  |
| --- |
|  |
|  |

3.1. Inflation breaths

3.2. Inserting of Oro-pharyngeal airway

3.3. Endo tracheal intubation

1. If the baby’s chest is not expanded after airway maneuvers, what would be you next intervention?

|  |
| --- |
|  |
|  |

4.1. Suctioning

4.2. Endo tracheal intubation

4.3. Start chest compression

1. If the baby’s heart rate is slow even after established baby’s breathing, what would you do?

|  |
| --- |
|  |
|  |

5.1. Give cardiac compression

5.2. Baby turn to left lateral position

5.3. Administer Adrenaline

1. If the mother has had Pethidine just before delivery and the baby is an apneoic with adequate ventilation, what would you do?

|  |
| --- |
|  |
|  |

6.1. Give Adrenaline

6.2. Give Sodium bicarbonate

6.3. Give Naloxone
